# Expression of p53 and p16 in Carcinoma Breast Tissue: Depicts Prognostic Significance or Coincidence

**DOI:** 10.7759/cureus.19395

**Published:** 2021-11-09

**Authors:** Manjit K Rana, Amrit Pal S Rana, Utkarshni Khera

**Affiliations:** 1 Pathology and Laboratory Medicine, All India Institute of Medical Sciences, Bathinda, IND; 2 General Surgery, All India Institute of Medical Sciences, Bathinda, IND

**Keywords:** immunohistochemistry, oncogenes, erbb2 protein, tumor suppressor protein p53, breast cancer

## Abstract

Breast cancer remains the most common malignancy among the Indian female population. The p16 and p53 genes are frequently mutated in breast cancer. Therefore, we aimed to evaluate the prognostic significance of p16 and p53 overexpression in breast cancer and their correlation with various traditional prognostic parameters. Total of 100 confirmed cases of breast cancers were selected. Patients who underwent chemotherapy treatment were excluded from the study. Estrogen receptor (ER), progesterone receptor (PR), and Her2neu immunohistochemistry were performed. The p16 and p53 immunohistochemistry was performed on all cases and association with various clinicopathologic parameters was determined. The mean age of carcinoma breast was 53.3+11.6 with age ranging from 28 to 82 years. On histopathological examination, 93% of cases were of invasive ductal cell carcinoma (IDC) with majority of grade I (43%). Only 14% of cases showed positive p53 expression and 19% of cases showed positive p16 expression. P16 was seen in a very significant correlation with p53 expression in all breast carcinoma cases (<0.002). p53 expression showed a positively significant (<0.05) correlation with age and grade III. The p16 expression was seen significantly correlated with low mitotic activity index (MAI) only. The p53 over-expression was seen in worse prognostic factors such as high tumor grade, Her2neu and triple-negative expression suggested its potential role in pathogenesis of carcinoma breast. In addition, high expression of p16 seen in low mitotic count and Her2neu expression also emphasized the role of this biomarker and recommends further molecular-based research.

## Introduction

Carcinoma breast is the most widespread malignant neoplasm among females globally. In India, breast cancer develops nearly a decade prior to the Western counterparts [1,2]. According to various pathological and biological characteristics of carcinoma patients diagnosed with the disease can present with different biological and clinical behavior. Estrogen receptor (ER) and progesterone receptor (PR) are the first molecular predictive markers to be used in carcinoma breast followed by HER2/neu [3]. In immunohistochemical (IHC) examination of the tumor tissue, breast cancer is categorized into luminal, HER2 positive and triple-negative (TN) [4-7]. However, despite the utilization of the effective therapies based on existing prognostic factors, recurrence may be seen in 20%-30% of breast cancer patients [8]. In India, as majority of the patients with breast cancer present in premenopausal age, though early-onset of breast cancer is associated with aggressive biological behaviour, so exploration of new prognostic indicators is further required [9]. However, to identify added parameters providing information about prognosis and therapeutic use in carcinoma breast is still a matter of controversy and research [4,10]. It has been analyzed that all the human cancers show dysregulated pathways of either p16 or p53. So, the p53 and p16 are important proteins involved in cell cycle and control pathways which are frequently targeted in human tumorigenesis [11]. The p53 and p16 overexpression can provide additional information about prognosis and the prognostic value of expression of mutation in p16 and p53 has been explored in carcinoma breast cancer in a variety of studies [12,13]. Yet studies done with p16 and p53 tumor expression in carcinoma breast among Indian patients are scanty. Hence, the association of p53 and p16 overexpression in carcinoma breast with various prognostic parameters like age, histopathological type, tumor grade, mitosis along with ER, PR and HER2neu expression was assessed in the present study. 

## Materials and methods

The retrospective study was conducted in a tertiary care cancer centre from 2018 to 2019 and 100 carcinoma breast cases who underwent core needle biopsy, breast conservative surgery and modified radical mastectomy were included. Patients who had taken chemotherapy treatment were excluded from the study. The hematoxylin and eosin (H&E)-stained histopathological sections were reviewed and age of the patients, histopathological grade, microscopic type and rate of mitosis were analyzed. As manual method for immunohistochemistry (IHC) technique was followed and to provide uniformity for antigen retrieval process, cores of the representative area from paraffin blocks were taken. Cores were re-embedded in paraffin wax and sections of cores were cut for H&E and IHC staining.

Tumors were histopathologically typified according to the World Health Organization (WHO), “Classification of Breast Tumors” [13]. IDC was classified according to well-differentiated (Grade I), moderately differentiated (Grade II), and poorly differentiated (Grade III). MAI was scored using light microscopy, mitosis was counted in 10 high power fields (40× magnification) or per unit area (2 mm^2^) in the most active part of the tumor [14].

**Figure 1:**
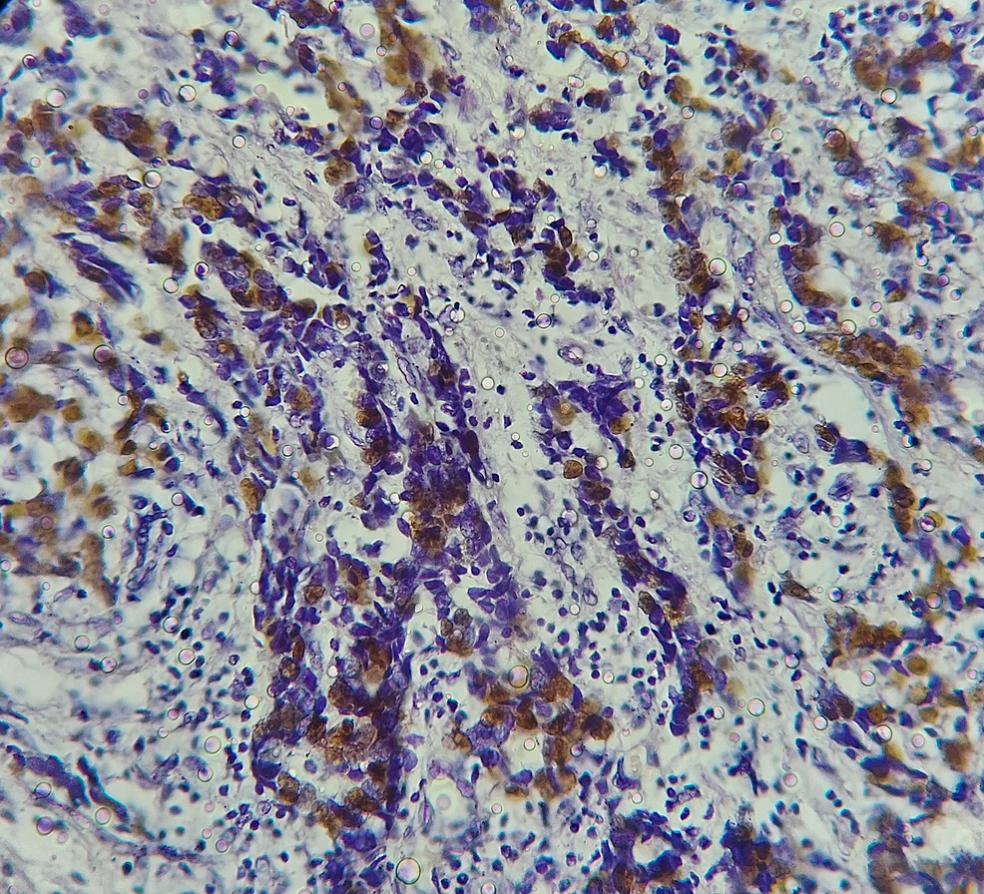
Strong IHC staining of p53 (40x). IHC: immunohistochemistry.

IHC for ER, PR, Her2neu, p16 and p53 were performed on sections of cores. Positive and negative controls were included routinely. Allred scoring was done for ER and PR interpretation. Whereas Her2- neu IHC staining was scored as per College of American Pathologist (CAP) guidelines into 1+ (weak), 2+ (intermediate) and 3+ (strong) expressions. [15,16] Expression of p53 in 5% of tumor cells was considered positive. Percentage of positive tumor cells was evaluated and scored as 0 for <5%, (1) for 5-25%, (2) for 25-50, (3) for 50-75 and 4 for >75.19,20 Staining intensity (SI) was considered as (1) for weak, (2) for medium and (3) for strong staining in evaluation of p53 (Figure 1) [6].

**Figure 2:**
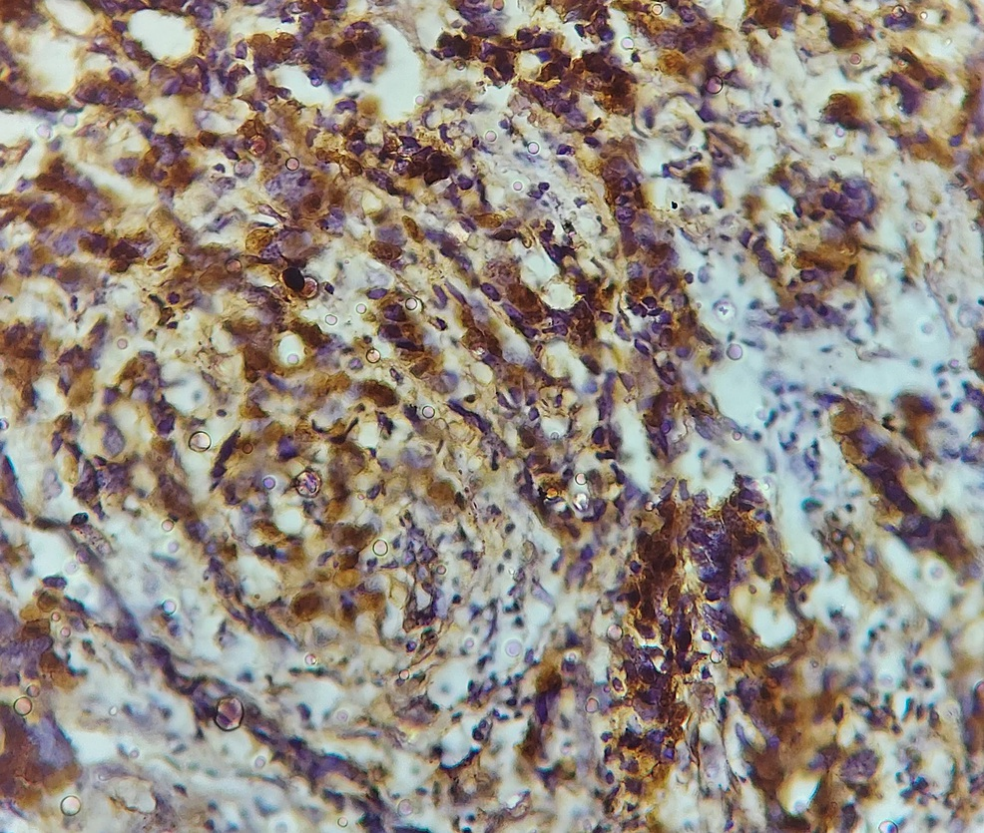
Strong nuclear and cytoplasmic IHC staining in case of p16 (40x). IHC: immunohistochemistry.

Immunohistochemical scores (IHCS) were calculated by multiplying percentage value with the staining intensity value. Both nuclear and cytoplasmic staining was evaluated in case of p16, whereas cytoplasmic staining alone was considered as negative (Figure 2).

Intensity of staining was categorized into no staining (0), weak (1+), intermediate (2+), strong (3+) while the percentage of positively stained cells were measured as a continuous variable. Intermediate to strong staining in > 10% cancer cells were considered positive while weak to intermediate staining in < 10% cancer cells was taken as focal positive. Cytoplasmic only staining, diffuse blush/weak intensity staining and other focal/patchy patterns were considered negative. [16]

Standard deviation (SD) and mean for frequency and percentage were evaluated for qualitative variables. P-value of ≤0.05 and ≤0.002 was taken as significant and very significant.

## Results

**Figure 3:**
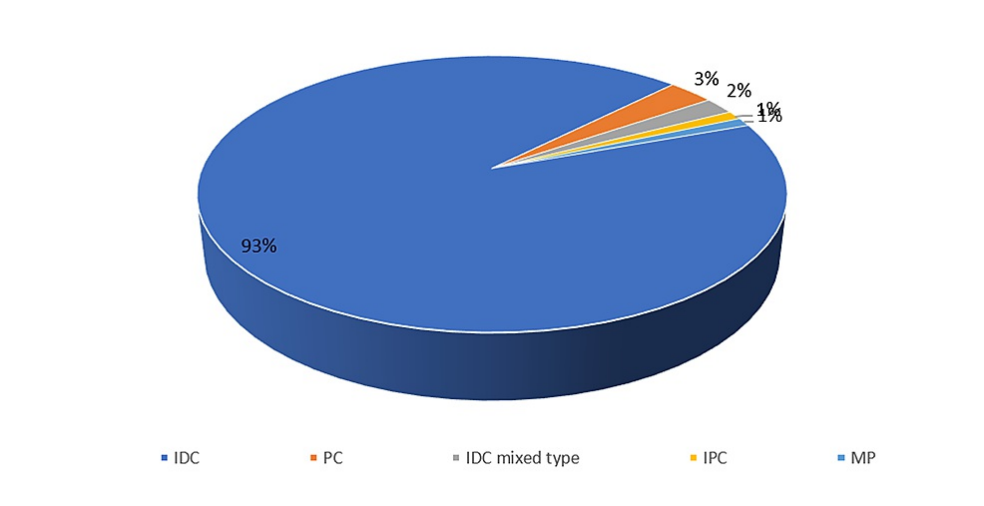
Distribution of histopathological type of carcinoma breast.

Out of 100 cases of carcinoma breast all patients were females with age ranging from 28 to 82 years with mean of 53.3+11.6. On histopathological examination, 93% of cases were of invasive ductal cell carcinoma (IDC) (Figure 3).

**Figure 4:**
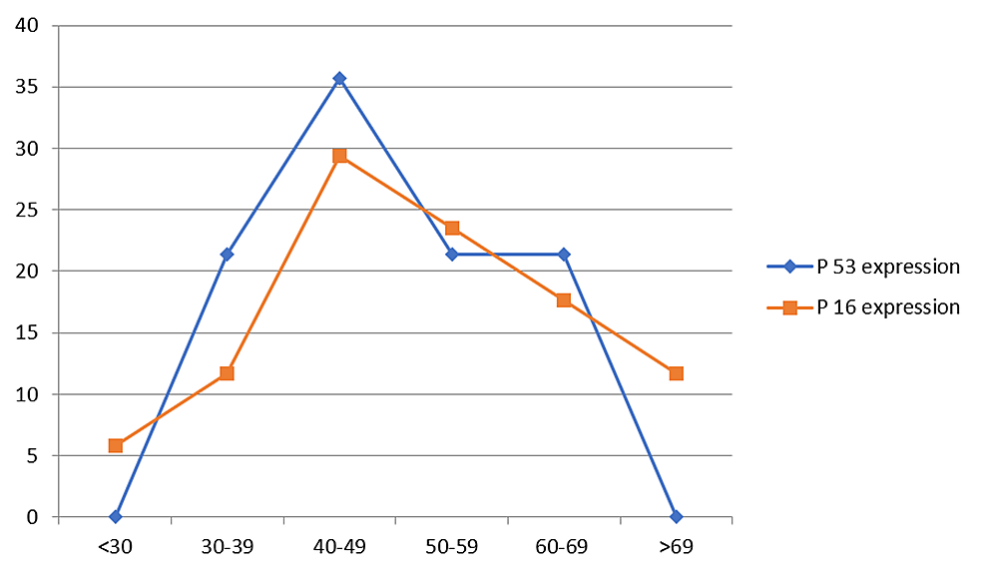
Correlation of age and p53, p16 expression.

And 43% were of grade I, 32% were of grade II and 25% were of grade III. Only 14% of cases showed positive p53 expression and 19% of cases showed positive p16 expression. p16 was seen in very significant correlation with p53 expression in all breast carcinoma cases (<0.002). P53 expression showed a positively significant (<0.05) correlation with age and grade III (Figure 4).

The p16 expression was seen significantly correlated with low MAI only (Table 1).

**Table 1:** MAI, p53 and p16 expression. MAI: mitotic activity index.

MAI	p53 expression	p16 expression
0-5	71.4%	82.3%
6-11	7.1%	17.6%
>11	21.4%	Nil
P-value	>0.05	<0.05

Only ER-positive and HER2neu cases showed positive correlation with p53 expression (Table 2).

**Table 2:** Correlation of tumor markers expression with p53 and p16.

ER+, PR+ status	HER2neu	p53	p16
ER+, PR+	-	Negative	Negative
ER+, PR-	-	42.8% (<0.05)	14.2%
ER-, PR+	-	Negative	Negative
ER+, PR+	+	Negative	Negative
ER-, PR-	+	80.0% (<0.002)	60%
ER-, PR-	-	3.8%	16.8%

## Discussion

Breast cancer is the most common cancer of females contributing approximately 11.7 % of all cancers with an estimated 2.3 million new cancer cases diagnosed in 2020 worldwide [1]. And carcinoma breast is the most common carcinoma with projected incidence of 1 in 29 females in the Indian population. The highest load of cancer breast was seen in the population of metropolitan cities. [5] In the present study, carcinoma breast was seen in the age of 28-82 years, with a mean age of 53.3 years. [4] As per data shown in GLOBOCON 2020 [1] A higher incidence rate has been observed in Asian countries in the younger age group in India and other Asian countries as compared to the western population. Due to lack of awareness and preventive measures and availability of treatment facilities both incidence and mortality of breast cancer is high in Indian females [5]. In Southeast Asia, carcinoma breast was seen associated with adverse prognostic features by Hashmi AA et al. [6]. Although the use of traditional immunomarkers has helped in developing targeted therapies for breast carcinoma. Rakha EA et al. [2] suggested that the molecular heterogeneity of carcinoma breast cannot be assessed morphologically and tumors classified with histological type and grade can present distinct molecular aspects and biological course. Hence carcinoma breast represents an important challenge for the research and treatment of breast cancer. Hormone receptors (ER and PR) and human epidermal growth factor receptor-2 (HER-2) are the most relevant clinical biomarkers that are widely used in stratifying breast cancer cases management and heterogeneity of the carcinoma breast and unpredictable behavior in many cases necessitate the exploration of prognostic use of other markers. In the current study group of carcinoma breast cases, 33% were triple-negative (TN). The prevalence of TN breast cancer in India varies between 27% to 35% with average estimation of 31 % [17]. Sen S et al. [7], Rao C et al. [8], also mentioned the more or less similar findings. In a review study done by Jha PK et al had mentioned that TN breast cancer is seen associated with bad prognosis and requires further research [18]. In the present study, p16 was seen insignificantly correlated in 16.8% of the cases whereas p53 showed expression in 3.8% only. However, Hashmi AA et al observed a significant correlation of p16 and p53 with TN breast carcinoma [19]. The different patterns of gene expression can differentiate breast carcinomas into four clinically relevant molecular subclasses such as Luminal A, Luminal B, enriched HER2 (HER2+), and Triple Negative (TN) [20]. As different subtypes of breast cancers may have specific mechanisms at molecular levels. The two major pathways involved in control of the cell cycle such as p16(INK4a) and p53 pathways depict the expression of tumor suppressor genes and are involved in human tumorigenesis [14,15,21,22]. There is significant evidence relating that p53 and p16 protein expression is associated with aggressive behavior and tumor proliferation of carcinoma breast. In the current study, high expression of p53 and p16 was noted in the age group 40-49 years. A work done by Li JP et al revealed high expression of p53 in a median age of 54 years [23]. The maximum positive correlation of p53 and p16 expression was seen with higher-grade breast cancer with significant association of p53. Overall, tumors with grade 3 on histopathological examination often showed positive immunoexpression for p53 and p16 most of which (50%) showed 3+ intense staining. Dang D et al. [11] emphasized the strong interactions between the p16 and p53 mutations in tumorigenesis. Tumors of histologic grade 1 or 2 also showed immunopositivity with p53. In concordance with our results, several reports in the literature have mentioned a statistically significant correlation between high histologic grade and p53 positivity [24-26]. The p16 expression was statistically correlated with lower mitotic rate tumors. In a work done by Shan M et al. [27] low levels of p16 expression were seen positively correlated higher proliferation index. Whereas, no substantial correlation of p53 with mitotic activity was recognized.

In the present study, various tumor markers expressions (ER+, PR+, HER2neu-), (ER- PR+, Her2 neu -) and (ER+, PR+, Her2 neu+) were seen negatively correlated with p 53 and p16. However, a strong positive correlation of p16 was observed in ER-, PR-, Her2 neu+ expression suggests the potential prognostic value of p16 in the carcinoma breast. In addition, higher expression of p53 was found to be positively significantly correlated with ER+, PR-, Her2 neu- and very significantly correlated with ER-, PR-, Her2 neu+ expression. Although P53 and P16 expressions showed variable expression in the tumor tissue but seen very significant correlated (<0.002) [6,28]. The limitations of our study were that no molecular testing of p16 and p53 was done thus has been suggested to find mutation status and correlation with IHC expression.

## Conclusions

The current work done showed a significant association of p53 IHC over-expression with high tumor grade and Her2nue expression and very significant association with p16 expression in the carcinoma breast tumor tissue. On the other hand, p16 expression was seen significantly correlated with low mitotic index and positively correlated with Her2neu expression. As p53 and p16 were seen significantly correlated with Her2neu expression, so can depict poor prognosis hence can be used as poor prognostic markers in carcinoma breast.
